# Overexpression of the miR-141/200c cluster promotes the migratory and invasive ability of triple-negative breast cancer cells through the activation of the FAK and PI3K/AKT signaling pathways by secreting VEGF-A

**DOI:** 10.1186/s12885-016-2620-7

**Published:** 2016-08-02

**Authors:** Sul Ki Choi, Hoe Suk Kim, Tiefeng Jin, Eun Hye Hwang, Minji Jung, Woo Kyung Moon

**Affiliations:** 1 Department of Radiology, Seoul National University Hospital, 101 Daehak-ro, Jongno-gu, Seoul 110-744 Korea; 2 Department of Biomedical Science, College of Medicine, Seoul National University, 103 Daehak-ro, Jongno-gu, Seoul 110-799 Korea

**Keywords:** Triple-negative breast cancer (TNBC), microrna-200 (miR-200), Vascular endothelial growth factor (VEGF), Migration, Invasion, Phosphatidylinositol-4,5-bisphosphate 3-kinase (PI3K), Protein kinase B (AKT), Focal adhesion kinase (FAK)

## Abstract

**Background:**

The role of microRNA-200 (miR-200) family members in the migration and invasion of breast cancer is controversial. This study investigated the mechanisms by which the miR-200 family members modulated the migratory and invasive abilities of an aggressive triple-negative breast cancer (TNBC) cell line, MDA-MB-231.

**Methods:**

The miR-200 family (miR-200b/200a/429 and miR-141/200c clusters) and green fluorescence protein (GFP) were transduced into MDA-MB-231 cells using a lentiviral system. Stable cells highly expressing the miR-200 family and GFP were isolated by puromycin selection and fluorescence-activated cell sorting. Gene expression was evaluated using real-time polymerase chain reaction (PCR) and reverse transcriptase-PCR (RT-PCR). The migratory and invasive abilities were assessed using trans-well and wound-healing assays. The secreted cytokines and growth factors in cultured media were quantified using a Bio-Plex200 multiplex array system. Western blot assays and immunofluorescence staining were conducted to investigate miR-200 family-regulated signaling pathways. The entire dataset obtained in this study was statistically evaluated using a one-way ANOVA followed by a *t*-test.

**Results:**

The stable overexpression of the miR-200b/200a/429 or miR-141/200c cluster suppressed cell growth and significantly increased migration and invasion of MDA-MB-231 cells. miR-141/200c overexpression was more effective in decreasing cell growth and promoting migration and invasion of MDA-MB-231 cells than was miR-200b/200a/429 overexpression. In addition, the overexpression of the miR-200b/200a/429 or miR-141/200c cluster led to an increase in the phosphorylation of focal adhesion kinase (FAK) and protein kinase B (AKT). Chemical inhibitors of FAK and phosphatidylinositol-4,5-bisphosphate 3-kinase (PI3K)/AKT suppressed the migration and invasion of MDA-MB-231 cells that was enhanced by the overexpression of the miR-200b/200a/429 or miR-141/200c cluster. Compared to the miR-200b/200a/429 cluster-transduced MDA-MB-231 cells, the miR-141/200c cluster-transduced MDA-MB-231 cells exhibited a significant increase in vascular endothelial growth factor (VEGF)-A secretion and integrin-alphaV (integrin-αV) expression. Treatment with an anti-VEGF-A-neutralizing antibody inhibited the increase in migration and invasion in both the miR-200b/200a/429- and miR-141/200c-transduced MDA-MB-231 cells but significantly reduced the phosphorylation of FAK and AKT in only the miR-141/200c cluster-transduced MDA-MB-231 cells.

**Conclusions:**

Taken together, our data demonstrate a mechanism in which the miR-141/200c cluster, through FAK- and PI3K/AKT-mediated signaling by means of increased VEGF-A secretion, promotes the migratory and invasive abilities of MDA-MB-231 cells.

**Electronic supplementary material:**

The online version of this article (doi:10.1186/s12885-016-2620-7) contains supplementary material, which is available to authorized users.

## Background

Aberrant expression of microRNAs (miRs), which are small non-coding RNA molecules consisting of approximately 22 nucleotides, has been identified in human cancer, where the miRNA signature is associated with specific clinical and biological features [1]. The microRNAs related to cancers may act as tumor suppressors or oncogenes, depending on the cancer type [2, 3]. The miR-200 family member genes are clustered at two locations in the genome: the miR-200b/200a/429 cluster and the miR-141/200c cluster [4]. The miR-200 family members repress the epithelial-to-mesenchymal transition (EMT), cancer cell migration, tumor growth, and metastasis by directly targeting specific genes, such as ZEB1, Suz12, moesin, and AP-2γ [4, 5]. In contrast, the miR-200 family members have been shown to enhance the migration ability of breast cancer cells and to promote the metastatic colonization of breast cancer cells through up-regulating the expression of E-cadherin and down-regulating that of ZEB2 and Sec23a [6, 7]. In a recent study, high expression of the miR-200 family was associated with a high probability of relapse, poor survival, and distant metastasis in breast cancer patients [8]. The loss of miR-200c expression has also been related to the induction of an aggressive, invasive, and chemoresistant phenotype of non–small cell lung cancer [9]. Conflicting results have been obtained in studies of the role of each miR-200 family member in repressing or enhancing cancer cell migration and invasion as well as the tumor growth and metastasis of diverse cancers, including breast cancer [10, 11].

Triple-negative breast cancer (TNBC) lacking estrogen receptor (ER), progesterone receptor (PR), and human epidermal growth factor receptor 2 (HER2) expression, is a highly invasive and metastatic form of breast cancer with a generally poorer prognosis than that of other breast cancer subtypes [12]. It is important to develop new treatment strategies based on a better understanding of the underlying mechanisms regulating the aggressive behavior of TNBCs. TNBCs express the miR-200 family members at a significantly lower level than do other subtypes of breast cancer, such as ER-positive or HER2-positive breast cancer [13]. Only a small number of the miR-200 target genes that are involved in breast cancer cell migration and metastasis have been identified [4–6], and few studies of the role of the miR-200b/200a/429 or miR-141/200c cluster in human TNBC have been conducted. The biological relevance of the function of the miR-200b/200a/429 or miR-141/200c cluster in human TNBC remains to be discovered.

Synthetic miR-200b directly downregulates vascular endothelial growth factor (VEGF) in endothelial cells and prevents the diabetes-induced increase in VEGF, thus inhibiting angiogenesis in diabetic retinopathy [14]. Chemokine CCL5 (formerly RANTES) of the CC-chemokine family, which plays a critical role in local invasion and distant metastasis in chondrosarcoma, promotes VEGF expression and angiogenesis by downregulating miR-200b [15]. The miR-200c radiosensitized the lung cancer cell line, A549 by targeting the VEGF-VEGFR2 pathway [16]. From these reports, we speculated that the overexpression of the miR-200 family can regulate the expression and secretion of cytokines and growth factors involving in cell growth and migratory and invasive abilities of TNBC cells.

In the present study, we used MDA-MB-231 cells, a typical human TNBC cell line, which were stably transduced with lentivirus carrying miR-200 family. We found that the overexpression of the miR-141/200c cluster promoted stronger migration and invasion as well as higher VEGF-A secretion in MDA-MB-231 cells. Therefore, we investigated, in detail, the mechanisms by which two miR-200 family members, the miR-200b/200a/429 cluster and the miR-141/200c cluster, regulated MDA-MB-231 cell migration and invasion. We demonstrate that the overexpression of the miR-141/200c cluster in MDA-MB-231 cells increased VEGF-A secretion, which enhanced the migratory ability of the cells through the activation of focal adhesion kinase (FAK) and the phosphatidylinositol-4,5-bisphosphate 3-kinase (PI3K)/protein kinase B (AKT) signaling pathway.

## Methods

### Cell culture

The human breast cancer cell lines MCF-7 (ER-positive subtype), MDA-MB-231 and HCC-38 (TN subtype) were obtained from the Korean Cell Line Bank (Seoul, Korea). Hs578T cells (TN subtype) were obtained from ATCC (Manassas, VA, USA). MCF-7 cells were grown in Dulbecco’s Modified Eagle’s Medium (DMEM) (WelGENE, Daegu, Korea) containing 10 % fetal bovine serum (FBS) and supplemented with a 1 % antibiotic solution containing penicillin and streptomycin (Gibco, Auckland, NZ). The MDA-MB-231, HCC-38, and Hs578T cells were grown in Roswell Park Memorial Institute (RPMI) 1640 medium (WelGENE) containing 10 % FBS and supplemented with a 1 % antibiotic solution containing penicillin and streptomycin (Gibco). The MCF-7, MDA-MB-231, HCC-38, and Hs578T cells used in this study were authenticated and validated by DNA fingerprinting (AmplFLSTR identifiler PCR Amplification kit), which was conducted by the Korean Cell Line Bank.

### Lentiviral transduction

Viral vectors containing either the miR-200b/200a/429 cluster (GenBank ID: 406984 406983) or the miR-141/200c cluster (GenBank ID: 406985 406933) constructs and the green fluorescent protein (GFP) construct were kindly supplied by Dr. Gregory J. Goodall of the University of Adelaide (Adelaide, Australia). A viral vector (pLenti M1.41) containing GFP was used as a control vector. Lentiviral transduction was conducted according to the manufacturer’s instructions. Briefly, cells were seeded at a density of approximately 10–25 % confluency (1 × 10^5^ cells) in 6-well plates and were maintained at 37 °C with 5 % CO_2_. Following an overnight culture, the culture medium was removed. Aliquots of the lentiviral stocks containing the miR-200b/200a/429 cluster or the miR-141/200c cluster construct as well as the control virus were gently mixed with 8 μg/ml polybrene and added to each well. After 6 h of transduction, the medium was replaced with fresh complete medium. Transduced cells with a cell density of greater than 90 % confluency were selected using medium containing 3 μg/ml puromycin for 2 weeks. Then, the GFP-positive cells were sorted from the selected cells using a FACSCalibur flow cytometer (BD Biosciences, Franklin Lakes, NJ, USA). Cancer cells stably expressing the miR-200b/200a/429 cluster or the miR-141/200c cluster and GFP, denoted as miR-200ab cells and miR-200c cells, respectively, and cancer cells containing the pLenti M1.41 vector, denoted as control cells, were generated and expanded for use in all subsequent studies.

### Quantitative real-time PCR

TaqMan MicroRNA Assays (Applied Biosystems, South San Francisco, CA, USA) were used to quantify the levels of mature miRNAs, following the manufacturer’s instructions. The miRNAs were isolated from cells using the mirVana miRNA isolation kit (Applied Biosystems), and the specific primers for detecting miR-200a, miR-200b, and, miR-200c were purchased from Applied Biosystems. Reverse transcription was performed using the TaqMan microRNA reverse transcription kit (Applied Biosystems) according to the manufacturer’s instructions. The traditional TaqMan Assay control, 18 s rRNA, was used as the endogenous control. Each TaqMan Assay was conducted in triplicate.

### Reverse transcriptase polymerase chain reaction (RT-PCR)

The total RNA was isolated using TRIzol Reagent (Invitrogen, Carlsbad, CA, USA) and reverse-transcribed using random hexamers and Superscript III reverse transcriptase. The cDNAs were synthesized using M-MLV reverse transcriptase (New England Biolabs, Ipswich, MA, USA) and random primers. The mRNA levels in the miR-200 family-transduced cells and non-transduced cells, referred to as the control, were evaluated using the conventional RT-PCR method with the following primer sets: E-cadherin (421 bp), F, ATTCTGATTCTGCTGCTCTTG and R, AGTAGTCATAGTCCTGGTCTT; Vimentin (247 bp), F, CCCTCACCTGTGAAGTGGAT and R, TCCAGCAGCTTCCTGTAGGT; ZEB1 (150 bp), F, TTCAAACCCATAGTGGTTGCT and R, TGGGAGATACCAAACCAACTG; ZEB2 (127 bp), F, CAAGAGGCGCAAACAAGC and R, GGTTGGCAATACCGTCATCC; Snail (557 bp), F, CAGACCCACTCAGATGTCAA and R, CATAGTTAGTCACACCTCGT; Fibronectin (171 bp), F, CAGAATCCAAGCGGAGAGAG and R, CATCCTCAGGGCTCGAGTAG; and β-actin (335 bp), F, TTCCTGGGCATGGAGTCCTGTGG, and R, CGCCTAGAAGCATTTGCGGTGG. Each target gene was amplified using a Thermocycler (BioRad, Hercules, CA, USA). The PCR products were subjected to electrophoresis through 1.5 % agarose gels, and the levels of gene expression were normalized to that of β-actin.

### Cell viability and proliferation assay

In vitro cell viability and proliferation were assessed using the 3-(4, 5-dimethylthiazol-2-yl)-2,5-diphenyl tetrazolium bromide (MTT) assay. Briefly, 5 × 10^3^ cells were allowed to adhere in a high-humidity environment in 5 % CO_2_ at 37 °C in 96-well culture plates. At 1 d, 3 d, and 5 d after cell seeding, the MTT solution (a final concentration of 1 mg/ml) was added, and the cells were incubated for 1 h. At the end of the incubation period, the MTT solution was carefully removed, and 150 μl of dimethyl sulfoxide was added to each well. The plates were maintained on a rocker shaker for 10 min at 25 °C, and then the amount of MTT formazan crystals formed by the viable cells was determined using a spectrophotometer based on the absorbance at 540 nm (GE Healthcare, Piscataway, NJ, USA). Flow cytometry analysis using 7-AAD was performed to confirm cell viability.

### Migration and invasion assay

To assess the cell migratory ability, 2–5 × 10^4^ cells were suspended in 100 μl of medium with or without 10 % FBS and deposited in the upper chambers of a trans-well plate with 8.0-μm pores (BD Biosciences) with a non-coated membrane. For the invasion assays, 5 × 10^4^ cells were plated in 2 % Matrigel™ (BD Biosciences) basement membrane matrix-coated upper chambers in a trans-well plate with 8.0-μm pores. The lower chambers were filled with 600 μl of medium supplemented with 10 % FBS, and the cells were incubated for 48 h at 37 °C in the presence or absence of an FAK inhibitor (5 μM PF573228, Sigma, St. Louis, MO, USA), a PI3K/AKT inhibitor (20 μM LY294002, Cell Signaling Technology, Danvers, MA, USA), 5 μg/ml VEGF-neutralizing antibody (Santa Cruz Biotechnology, Dallas, TX, USA) or 10 ng/ml VEGF-A protein (Sigma). Each inhibitor, VEGF-neutralizing antibody, or VEGF-A were added in both the upper and lower chambers. No chemoattractants were used in the lower chamber for either the migration or invasion assays. The cells that migrated from the upper chamber were stained using a crystal violet solution (0.5 % crystal violet in 20 % methanol) for 5 min. Unbound crystal violet was removed by rinsing using distilled water. The cells were subsequently air-dried, and the crystal violet was eluted from the cells using a solution of 1 % sodium dodecyl sulfate (SDS). The absorbance of crystal violet at 550 nm was measured using a spectrophotometer (GE Healthcare).

### Wound-healing assay

Cells were seeded at 5 × 10^4^ cells per well in six-well plates and cultured under permissive conditions until reaching 90 % confluence. After 24 h, each confluent cell monolayer was lightly and quickly scratched using a sterile plastic tip to produce a straight line. The debris was removed, and the edge of the scratch was smoothed by washing with PBS. The cells were cultured for 6 h in complete medium, after which, the lateral migratory activity was evaluated based on the area occupied by the cells that had entered the scratch line at 0 h. Images were acquired using a microscope (Leica, Wetzlar, Germany) equipped with a CCD camera (Leica). The migration rates were calculated according to the equation percentage wound healing = {(wound length at 0 h) - (wound length at 6 h)} / (wound length at 0 h) × 100. The mean results of three straight distances (upper edge, middle, and lower edge) in scratch area were evaluated as wound lengths. Length quantification was performed using ImageJ software (NIH, Bethesda, MD, USA).

### Measurement of cytokines and growth factors

Samples of 1–2 × 10^5^ cells were seeded in 6-well plates. After a two-day culture period, when cells were at 90 % confluency, the conditioned medium was harvested. The levels of secreted cytokines and growth factor (IL-2, IL-4, IL-5, IL-10, IL-12, IL-13, GM-CSF, IFN--γ, and VEGF-A) were quantified using the Bio-Plex200 multiplex array system according to the recommended protocol (Bio-Rad). All samples and standardized solutions were analyzed in triplicate.

### Western blotting

The cells were lysed in RIPA buffer (Sigma). The proteins were separated using SDS-polyacrylamide gel electrophoresis and were transferred to nitrocellulose membranes. The membranes were blocked using 5 % skim milk in Tris-buffered saline containing Tween and incubated with primary antibodies directed against ERK, phospho-ERK, AKT (anti-rabbit polyclonal antibody, Cell Signaling Technology), phospho-AKT (anti-mouse polyclonal antibody, Cell Signaling Technology), FAK (anti-rabbit polyclonal antibody, Invitrogen), phospho-FAK (anti-rabbit polyclonal antibody, Invitrogen), integrin-αV (anti-rabbit polyclonal antibody, Santa Cruz Biotechnology) or β-actin (anti-mouse polyclonal antibody, Sigma) overnight at 4 °C. The membranes were then incubated with horseradish peroxidase-conjugated secondary antibodies (Santa Cruz Biotechnology). The blotted membranes were visualized using enhanced chemiluminescence reagents (GE Healthcare). Western blot quantification was performed using ImageJ software.

### Immunofluorescence staining

The cells were seeded on sterile cover slips in 24-well plates. Then, the cells were fixed using a 4 % paraformaldehyde solution (Affymetrix, Cleveland, OH, USA). Primary antibodies directed against integrin-αV (Santa Cruz Biotechnology) and phospho-FAK (Invitrogen) were applied overnight at 4 °C. The bound integrin-αV and phospho-FAK antibodies were visualized using secondary antibodies conjugated to Alexa 594, and the nuclei were counterstained using 4',6-diamidino-2-phenylindole (DAPI). All multicolor fluorescence images were obtained using a confocal laser-scanning microscopy (LSM5 Meta) (Carl Zeiss, Oberkochen, Germany).

### Statistical analyses

For the entire dataset obtained in this study, the mean values ± standard deviations were calculated from the results of at least three independent experiments and were statistically evaluated using a one-way ANOVA followed by the *t*-test. For all tests, p-values of less than 0.05 were considered significant.

## Results

### Overexpression of the miR-200b/200a/429 cluster or the miR-141/200c cluster enhanced the migratory and invasive abilities of MDA-MB-231 cells

**Fig. 1:**
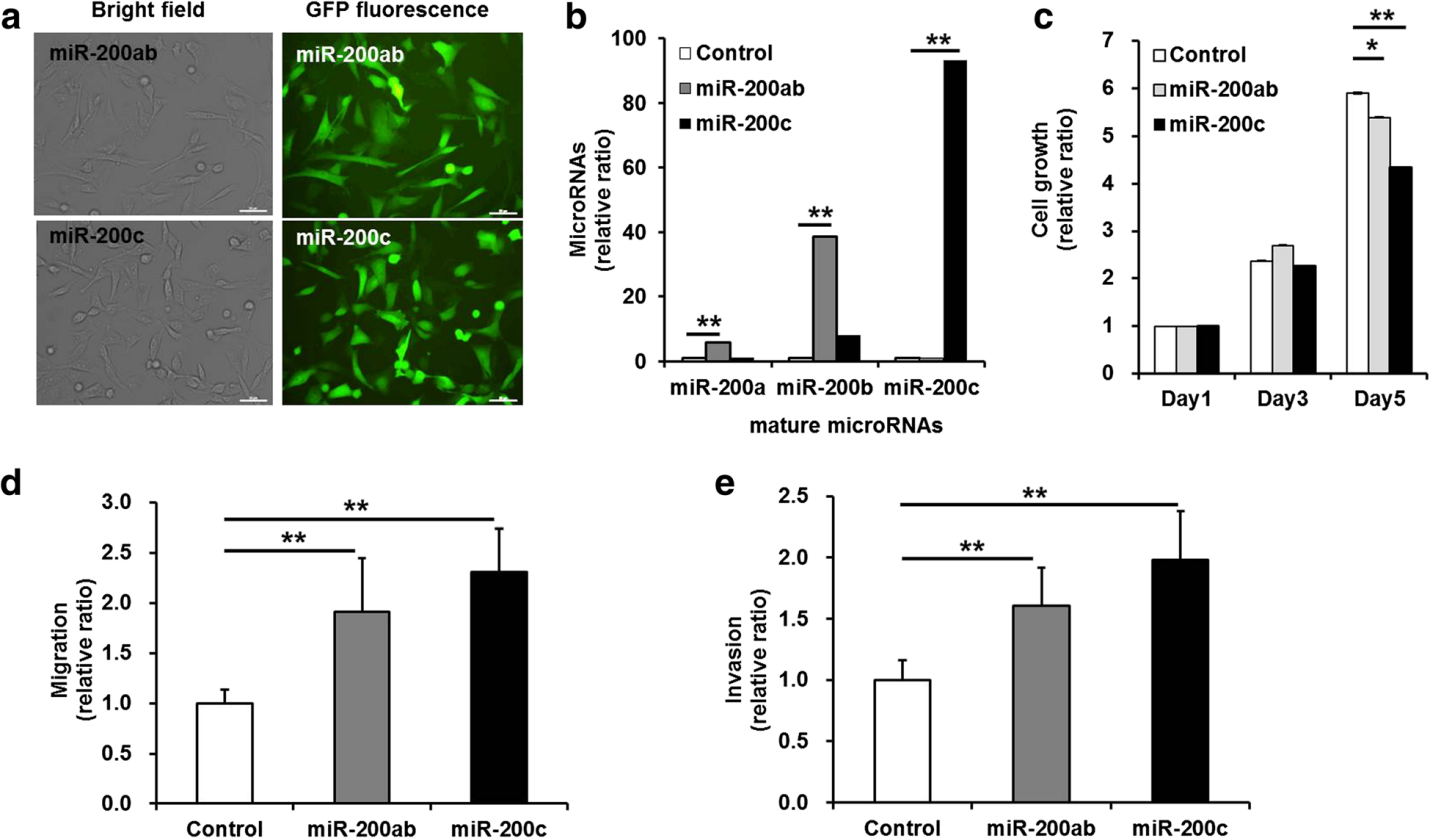
Proliferation, migration, and invasion of miR-200b/200a/429- or miR-141/200c-transduced MDA-MB-231 cells. **a** Fluorescence images of green fluorescent protein in MCF-7 and MDA-MB-231 cells that were transduced using lentivirus encoding both GFP and miR-200 family members. Strong GFP expression was detected in the miR-200 family-transduced cells. Scale bar; 50 μm. **b** Quantitative real-time RT-PCR of microRNAs (miR-200a, miR-200b, and miR-200c). The miR-200ab cells transduced with the miR-200b/200a/429 cluster construct exhibited high expression levels of miR-200a (~6-fold) and miR-200b (~40-fold) relative to those of the control cells. The miR-200c cells transduced with the miR-200c/141 cluster construct exhibited remarkably high expression levels of miR-200c (~93-fold) and miR-200b (~8-fold) relative to those of the control cells. **c** MTT assay for analysis of cell proliferation. The growth rate was significantly lower in the miR-200c cells (~4-fold) than in the control cells (~6-fold) at the 5th day. **d** Trans-well migration assay for the analysis of cell migration. **e** Trans-well invasion assay for the analysis of the invasive capacity. The migratory and invasive abilities of the miR-200ab and miR-200c cells (~2-fold) were significantly increased compared with those of the control cells. The enhanced migratory and invasive abilities were higher in miR-200c cells than in miR-200ab cells. All experiments were performed at least in triplicate, and the values are the mean values ± standard deviation. *p < 0.05, **p < 0.001

We first investigated alterations in the characteristics of two different breast cancer cell lines, MCF-7 and MDA-MB-231 cells, caused by the stable transduction of constructs encoding miR-200 family members and GFP using lentiviruses. The GFP-positive cells comprised more than 95 % of the miR-200b/200a/429 cluster- or miR-141/200c cluster-transduced MCF-7 and MDA-MB-231 cells (Additional file 1: Figure S1 and Additional file 2: Figure S2). Strong GFP expression in the miR-200b/200a/429 cluster- or miR-141/200c cluster-transduced MCF-7 and MDA-MB-231 cells was evaluated using microscopy (Fig. 1a and Additional file 1: Figure S1A). RT-PCR was used to evaluate expression levels of EMT markers, and the results showed that the overexpression of the miR-200b/200a/429 cluster or the miR-141/200c cluster resulted in the induction of E-cadherin expression and a decrease in ZEB-1 expression in MDA-MB-231 cells (Additional file 1: Figure S1B). Overexpression of the miR-200b/200a/429 or miR-141/200c cluster led to increased expression of Snail in MCF-7 and MDA-MB-231 cells.

To investigate the stable overexpression of miR-200a, miR-200b, miR-200c in miR-200b/200a/429 cluster- or miR-141/200c cluster-transduced MDA-MB-231 cells, real-time RT-PCR was conducted to quantify the levels of the mature microRNAs. As expected, the miR-200b/200a/429 cluster- and miR-141/200c cluster-transduced MDA-MB-231 cells showed increased mature miR-200a, miR-200b, and miR-200c levels at 5-100-fold higher levels than did the control MDA-MB-231 cells, which exhibited undetectable levels of all members of the miR-200 family (Fig. 1b).

To investigate whether overexpression of the miR-200b/200a/429 or miR-141/200c cluster affected cell growth, an MTT assay was performed. Figure 1c shows that the growth rate of the miR-200b/200a/429 cluster- or miR-141/200c cluster-transduced MDA-MB-231 cells was similar to that of the control cells on the 3rd day but was significantly decreased on the 5th day after cell seeding compared with that of the control cells (control vs. miR-200ab, *p* = 0.02 and control vs. miR-200c, *p* = 0.0002) (Fig. 1c). The overexpression of the miR-141/200c cluster was found to more strongly suppress the growth of MDA-MB-231 cells.

We assessed migratory and invasive abilities using a trans-well migration assay and a wound-healing assay. The results of crystal violet staining showed that the migratory ability of MCF-7 cells was suppressed by transduction of the miR-200 family, but there was no significant difference between that of the miR-200b/200a/429 cluster- or miR-141/200c cluster-transduced MCF-7 cells and control cells (Additional file 1: Figure S1C, E). The wound-healing assay, which demonstrated the lateral migratory ability of MCF-7 cells, yielded results similar to those of the trans-well migration assay (Additional file 1: Figure S1D, F).

With regard to the trans-well migration and invasion assays of the miR-200b/200a/429 cluster- or miR-141/200c cluster-transduced MDA-MB-231 cells, after 48 h of incubation without an FBS gradient, cell migration significantly increased up to 1.94 ± 0.22-fold and 2.49 ± 0.08-fold in miR-200b/200a/429 cluster- or miR-141/200c cluster-transduced MDA-MB-231 cells, respectively, compared with control cells (Fig. 1d control vs. miR-200ab, *p* = 0.04 and control vs. miR-200c, *p* = 0.002). To further investigate the migratory ability of miR-141/200c cluster-transduced MDA-MB-231 cells, the trans-well migration assay under the condition of a 10 % FBS gradient in the upper (0 % FBS) and the lower chamber (10 % FBS) was conducted for 24 h. A significant increase was observed in the migratory ability of the miR-141/200c-transduced MDA-MB-231 cells (1.53 ± 0.30-fold) relative to MDA-MB-231 cells (Additional file 3: Figure S3, *p* = 0.0009). As expected, the results of an invasion assay using Matrigel™ matrix-coated trans-well membranes showed a significant increase in the invasive ability of the miR-200b/200a/429 cluster-transduced cells (1.61 ± 0.31-fold) and miR-141/200c cluster-transduced cells (1.98 ± 0.40-fold) after 48 h of incubation compared with that of control cells (Fig. 1e, control vs. miR-200ab, *p* = 0.0005 and control vs. miR-200c, *p* = 0.0002). The elevated migration and invasion rates were higher in miR-141/200c cluster-transduced MDA-MB-231 cells than miR-200b/200a/429 cluster-transduced MDA-MB-231 cells. After 6 h of incubation, the transduction of miR-200b/200a/429 cluster or miR-141/200c cluster did not affect the lateral migratory ability of MCF-7 cells, but increased the lateral migration up to 2.78 ± 0.11-fold and 1.69 ± 0.11-fold in miR-200b/200a/429 cluster- or miR-141/200c cluster-transduced MDA-MB-231 cells, respectively (*p* = 0.02, Additional file 1: Figure S1F). In other TNBC cell lines, HCC-38 and Hs578T cells, we observed a significant increase in trans-well migration ability in both the miR-141/200c cluster-transduced HCC-38 cells (1.64 ± 0.11-fold, *p* < 0.001) and miR-141/200c cluster-transduced Hs578T cells (1.76 ± 0.44-fold, *p* = 0.0003) compared with the control cells (Additional file 4: Figure S4A, D). The mature miR-200c levels in miR-141/200c cluster-transduced HCC-38 and Hs578T cells were 252- and 205-fold higher than that in the control HCC-38 and Hs578T cells, which exhibited undetectable levels of miR-200c (*p* < 0.001, Additional file 5: Figure S5G, H).

We found that the overexpression of the miR-200b/200a/429 cluster or the miR-141/200c cluster resulted in the highest migratory capacity in MDA-MB-231 cells compared with the other TNBC cells, HCC-38 and Hs578T cells. Therefore, we focused on the regulatory mechanisms by which the miR-200b/200a/429 cluster or the miR-141/200c cluster promoted the migratory and invasive capacities of the MDA-MB-231 cell line, as a representative TNBC cell line.

### Overexpression of the miR-200b/200a/429 cluster or the miR-141/200c cluster enhanced the phosphorylation of FAK and AKT and the expression of integrin in MDA-MB-231 cells

**Fig. 2:**
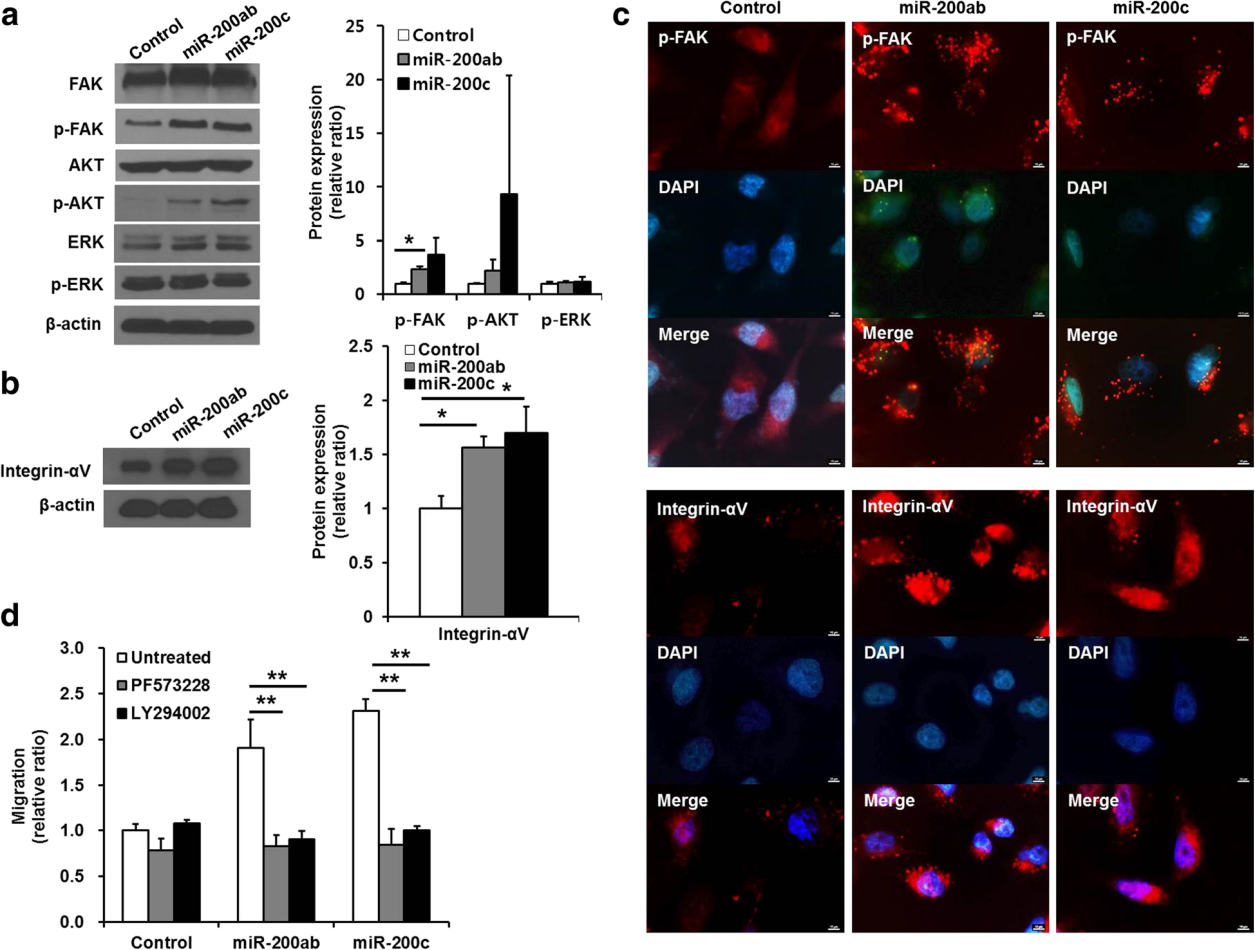
Signaling pathway associated with enhanced migration in miR-200b/200a/429 or miR-141/200c-transduced MDA-MB-231 cells. **a, b** Western blotting analysis of the levels of phosphorylated FAK, AKT, ERK, and integrin-αV expression. The levels of FAK and AKT phosphorylation were significantly higher in the miR-200 family-transduced cells than in the control cells, but the level of ERK phosphorylation was similar between the miR-200 family-transduced cells and control cells. The miR-200 family-transduction also increased integrin-αV expression. Densitometric quantifications of FAK, AKT, and ERK phosphorylation in the miR-200 family-transduced cells relative to control cells. β − actin was used as an internal reference. **c** Immunofluorescence analysis of phosphorylated FAK and integrin-αV. Phosphorylated FAK and integrin-αV were overexpressed and were highly localized at membrane surfaces in the miR-200 family-transduced cells. **d** Trans-well migration assay of FAK (PF573228) and PI3K/AKT (LY294002)-inhibitor-treated cells. Treatment with inhibitors of the FAK and PI3K/AKT signaling pathways significantly suppressed the cell migration enhanced by the transduction of miR-200 family. All experiments were performed at least in triplicate, and the values are the mean values ± standard deviation. *p < 0.05 **p < 0.001, Scale bar, 10 μm

We investigated whether the overexpression of miR-200 family members modulated the focal adhesion kinase (FAK), PI3K/AKT, and MEK/ERK signaling pathways, which are involved in cell proliferation and migration. Representative western blotting results showed that the levels of FAK and AKT phosphorylation were greatly increased, but the level of ERK phosphorylation was not significantly changed in the miR-200b/200a/429 cluster- or miR-141/200c cluster-transduced MDA-MB-231 cells (Fig. 2a, left). The level of phosphorylated FAK was significantly increased up to 1.52 ± 0.26-fold and 3.12 ± 1.56-fold in the miR-200b/200a/429 cluster-transduced cells and miR-141/200c cluster-transduced cells, respectively (Fig. 2a, right, control vs. miR-200ab, *p* = 0.01 and control vs. miR-200c, *p* = 0.09). The level of phosphorylated AKT was also increased 2.21 ± 1.06-fold and 9.32 ± 11.07-fold in the miR-200b/200a/429 cluster-transduced cells and miR-141/200c cluster-transduced cells, respectively (Fig. 2a, right, control vs. miR-200ab, *p* = 0.19 and control vs. miR-200c, *p* = 0.32). We also observed that the miR-141/200c cluster significantly increased the phosphorylation levels of FAK (1.98 ± 0.37-fold, *p* = 0.04) and AKT (5.61 ± 1.73-fold, *p* = 0.04) in HCC-38 cells (Additional file 6: Figure S6C, D). Representative western blotting results showed that the expression levels of integrin-αV, which is associated with the FAK signaling pathway, were increased in both the miR-200b/200a/429 cluster- and miR-141/200c cluster-transduced cells (Fig. 2b, left). Integrin-αV expression was increased 1.57 ± 0.10-fold and 1.70 ± 0.24-fold in the miR-200b/200a/429 cluster-transduced cells and miR-141/200c cluster-transduced cells, respectively (Fig. 2b, right, control vs. miR-200ab, *p* = 0.01 and control vs. miR-200c, p = 0.04). Integrin-αV expression was also increased (13.61 ± 0.72-fold, *p* = 0.008) in miR-141/200c cluster-transduced HCC-38 cells (Additional file 6: Figure S6C, D). To investigate the cellular localization of phosphorylated FAK and integrin-αV, immunofluorescent staining was assessed. Phosphorylated FAK was largely localized at the focal adhesions of the plasma membranes of the miR-200b/200a/429 cluster- and miR-141/200c cluster-transduced cells (Fig. 2c). Integrin-αV accumulated and clustered at the periphery of the plasma membranes of the miR-200b/200a/429- and miR-141/200c-transduced cells compared with that in control cells (Fig. 2c). The levels of FAK phosphorylation and integrin-αV expression were higher in the miR-141/200c cluster-transduced HCC-38 cells than in the control cells (Additional file 6: Figure S6E).

To determine the signaling pathway involved in enhanced migratory and invasive ability of miR-200b/200a/429 cluster- or miR-141/200c cluster-transduced MDA-MB-231 cells, trans-well migration assays were performed in the presence of PF573228 or LY294002, which are chemical inhibitors of FAK and PI3K/AKT, respectively. The chemical inhibitors were added to both the upper and lower chambers, which would allow their effects to last throughout the entire experimental period. The enhanced migratory activity was completely inhibited in miR-200b/200a/429 cluster- and miR-141/200c cluster-transduced MDA-MB-231 cells treated with the FAK or PI3K/AKT inhibitor, reaching the basal migratory level of MDA-MB-231 cells (Fig. 2d). These observations demonstrated that the elevated migratory ability of MDA-MB-231 cells by stable overexpression of miR-200b/200a/429 cluster or miR-141/200c cluster was driven by the activation of the FAK and PI3K/AKT-mediated signaling pathways.

### Overexpression of the miR-200b/200a/429 cluster or the miR-141/200c cluster increased the level of VEGF-A secretion in MDA-MB-231 cells

**Fig. 3:**
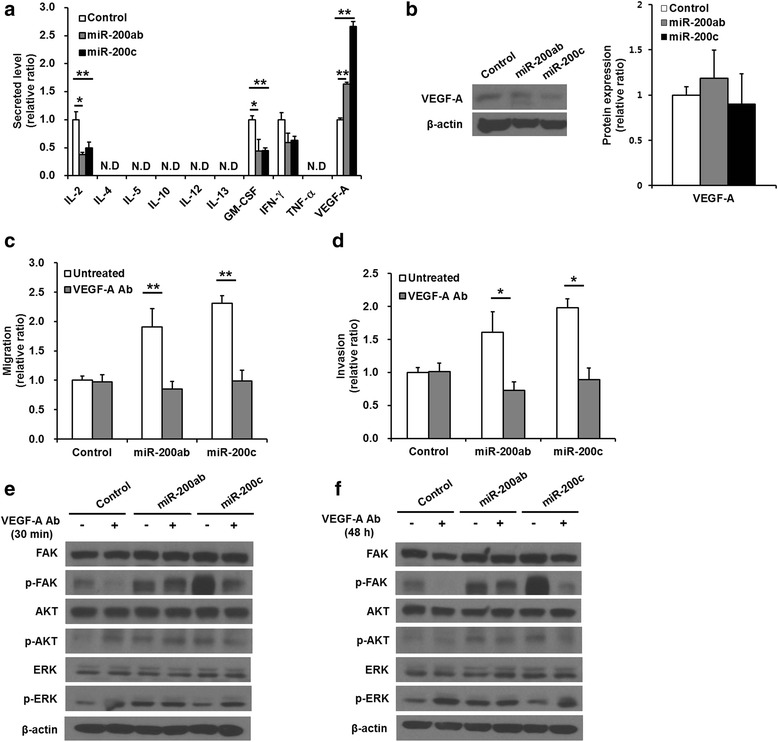
Migration and invasion in miR-200b/200a/429 or miR-141/200c-transduced MDA-MB-231 cells treated with an anti-VEGF-A-neutralizing antibody. **a** Measurement of the secreted levels of cytokines and growth factors (IL-2, IL-4, IL-5, IL-10, IL-12, IL-13, GM-CSF, IFN-γ, TNF-α, and VEGF-A). Transduction of the miR-200 family into MDA-MB-231 cells promoted significantly higher VEGF-A secretion than that of control cells. **b** Western blotting analysis of the levels of VEGF-A. **c** Trans-well migration and **d** invasion assay of anti-VEGF-A-neutralizing antibody-treated cells. The enhanced migration and invasion of the miR-200 family-transduced cells were significantly suppressed by treatment with anti-VEGF-A-neutralizing antibodies. **e, f** Representative image of western blotting of phosphorylated AKT, FAK, and ERK and total AKT, FAK and ERK in cells treated with anti-VEGF-A-neutralizing antibodies for 30 min and 48 h. The elevated phosphorylation levels of FAK and AKT in only miR-141/200c cluster-transduced cells but not miR-200b/200a/429-transduced cells were decreased by the anti-VEGF-A-neutralizing antibody, whereas the phosphorylation level of ERK was increased in miR-141/200c -transduced cells and control cells treated with anti-VEGF-A-neutralizing antibodies. All experiments were performed at least in triplicate, and the values are the mean values ± standard deviation. N.D: not detectable. *p < 0.05, **p < 0.001

The secreted cytokines or growth factors that might be involved in regulating the migration of the miR-200b/200a/429 cluster- or miR-141/200c cluster-transduced cells were analyzed. The levels of IL-2, GM-CSF, and IFN-γ secreted into the medium by the miR-200b/200a/429 cluster- and miR-141/200c cluster-transduced MDA-MB-231 cells after 48 h of culture were lower than those of the control MDA-MB-231 cells. However, the secreted VEGF-A levels were significantly higher in the conditioned medium of the miR-200b/200a/429 cluster-transduced cells (1.64 ± 0.03-fold) and miR-141/200c cluster-transduced cells (2.66 ± 0.09-fold) than in that of the control MDA-MB-231 cells (Fig. 3a, control vs. miR-200ab, *p* = 0.007 and control vs. miR-200c, *p* = 0.0007). Increased secretion of VEGF-A was higher in miR-141/200c cluster-transduced cells than miR-200b/200a/429 cluster-transduced cells (miR-200ab vs. miR-200c, *p* = 0.0001). The secreted levels of cytokines and growth factors were also evaluated in the miR-141/200c cluster-transduced HCC-38 and Hs578T cells. VEGF-A secretions were significantly increased in miR-141/200c cluster-transduced HCC-38 (1.75 ± 0.03-fold, *p* = 0.00001) and miR-141/200c cluster-transduced Hs578T cells (1.17 ± 0.16-fold, *p* = 0.004) relative to control cells (Additional file 4: Figure S4B, E). The effect of miR-141/200c overexpression on increased cell migration and VEGF-A secretion was greater in MDA-MB-231 cells relative to HCC-38 and Hs578T cells. There were no definite differences in VEGF-A secretion levels between the miR-141/200c cluster–transduced Hs578T and the control Hs578T cells. These results were explained by the phenotypic and functional heterogeneity among cancer cells in TNBC cells. The VEGF-A expression levels were also examined in the miR-200b/200a/429 cluster- or miR-141/200c cluster-transduced MDA-MB-231 cells, but the transduction of miR-200b/200a/429 cluster (1.18 ± 0.31-fold) or miR-141/200c cluster (0.90 ± 0.34-fold) did not affect VEGF-A expression (Fig. 3b, right, control vs. miR-200ab, *p* = 0.38 and control vs. miR-200c, *p* = 0.45). The miR-141/200c cluster–transduced HCC-38 cells (0.92 ± 0.09-fold) also expressed VEGF-A levels that were similar to the control HCC-38 cells (Additional file 6: Figure S6A, B, *p* = 0.46).

### Blocking VEGF-A activity inhibited the enhanced migration and invasion of miR-200b/200a/429 cluster or miR-141/200c cluster-transduced MDA-MB-231 cells

To examine whether secreted VEGF-A regulates the migratory and invasive abilities of the miR-200b/200a/429 cluster- or miR-141/200c cluster-transduced MDA-MB-231 cells, trans-well migration and invasion assays were performed after treatment with anti-VEGF-A-neutralizing antibodies. The anti-VEGF-A-neutralizing antibodies were added to both the upper and lower chamber, which would allow their effects to last throughout the entire experimental period. Figure 3c, d d showed that the anti-VEGF-A-neutralizing antibodies completely inhibited the increased migratory (control vs. miR-200ab, *p* = 0.0001 and control vs. miR-200c, *p* = 0.002) and invasive (control vs. miR-200ab, *p* = 0.02 and control vs. miR-200c, *p* = 0.001) abilities of the miR-200b/200a/429 cluster- and miR-141/200c cluster-transduced cells. These data clearly indicated that secreted VEGF-A was involved in promoting the migration and invasion of both miR-200b/200a/429 cluster- and miR141/200c cluster-transduced cells. The administration of anti-VEGF-A-antibodies for neutralizing VEGF-A in cultured medium suppressed the migration increased by miR-141/200c overexpression in both the miR-141/200c cluster-transduced HCC-38 and miR-141/200c cluster-transduced Hs578T cells (Additional file 4: Figure S4C, F). The anti-VEGF-A-neutralizing antibodies also completely inhibited the increased migratory ability (control vs. miR-200c, *p* < 0.001) of the miR-141/200c cluster-transduced HCC-38 cells (Additional file 4: Figure S4C, untreated vs. VEGF-A Ab, *p* < 0.001). These results supported that VEGF-A secretion was associated with enhancing migration ability in TNBC cells. The anti-VEGF-A-neutralizing antibodies partly blocked the enhanced migratory ability (control vs. miR-200c, untreated, 1.76 ± 0.44-fold, *p* = 0.0003) of both the control (0.80 ± 0.07-fold, *p* = 0.003) and the miR-141/200c cluster-transduced Hs578T cells (1.37 ± 0.21-fold, *p* = 0.003), but the miR-141/200c cluster-transduced Hs578T cells still showed increased migratory ability compared with that of the control cells (control vs. miR-200c, VEGF-A Ab treated, *p* < 0.001, Additional file 4: Figure S4F). These results imply that other factors besides VEGF-A may also be involved in promoting migration in miR-141/200c cluster-transduced Hs578T cells.

### The miR-141/200c cluster activated the FAK and PI3K/AKT signaling pathway by secreted VEGF-A, resulting in the promotion of the migratory and invasive abilities of MDA-MB-231 cells

To ascertain the VEGF-A-mediated intracellular signaling pathway responsible for the increased migratory and invasive abilities of miR-200b/200a/429 cluster- or miR141/200c cluster-transduced MDA-MB-231 cells, the levels of FAK and AKT phosphorylation were evaluated in cells treated with 5 μg/ml anti-VEGF-A-neutralizing antibodies. Treatment with anti-VEGF-A-neutralizing antibodies for both 30 min and 48 h remarkably decreased the level of FAK and AKT phosphorylation in the miR-141/200c cluster-transduced MDA-MB-231 cells and the control cells but did not affect the level of phosphorylated FAK and AKT in the miR-200b/200a/429 cluster-transduced MDA-MB-231 cells (Fig. 3e, f ). In contrast, the level of ERK phosphorylation was increased by anti-VEGF-A-neutralizing antibodies in miR-141/200c-transduced cells and control cells. These data implied that FAK and AKT acted as a direct link between elevated VEGF-A secretion and the consequential signal transduction related to enhanced migration of miR-141/200c cluster-transduced MDA-MB-231 cells, but the increased secretion of VEGF-A in miR-200b/200a/429 cluster-transduced MDA-MB-231 cells was not directly followed by activation of the FAK and AKT-mediated signaling pathway, which was possibly induced by the other signaling pathways.

### Exogenous VEGF-A stimulated migration of MDA-MB-231 cells by activating the FAK and PI3K/AKT pathway

**Fig. 4:**
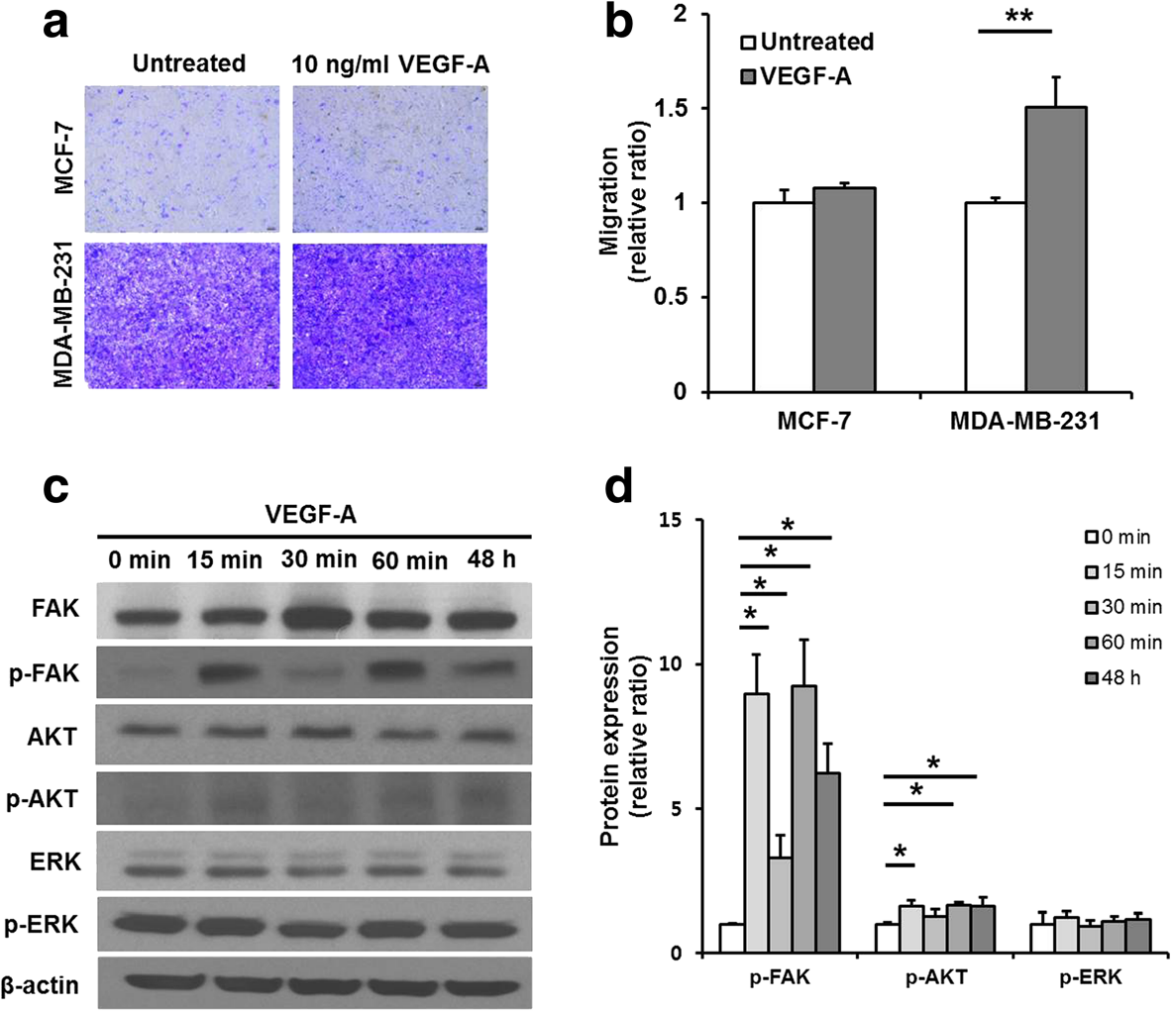
Migration and signaling pathways in VEGF-A-stimulated MDA-MB-231 cells. **a** Images of the crystal violet-stained cells that migrated horizontally in the trans-well migration assay. **b** The absorbance values of extracted crystal violet in migrated cells. Migration of the VEGF-A-treated MDA-MB-231 cells (~1.5-fold) was enhanced compared with that of the untreated control cells. **c** Representative image of western blotting of phosphorylated AKT, FAK and ERK and total AKT, FAK and ERK in MDA-MB-231 cells treated with VEGF-A. The levels of FAK and AKT phosphorylation were increased in the VEGF-A-treated MDA-MB-231 cells, but the level of ERK phosphorylation was similar between VEGF-A-treated MDA-MB-231 cells and the untreated cells. **d** Densitometric quantifications of phosphorylation levels of FAK, AKT, and ERK in the VEGF-A-treated cells relative to the untreated cells. β−actin was used as an internal reference. All experiments were performed at least in triplicate, and the values are the mean values ± standard deviation. *p < 0.05, **p < 0.001

To confirm the role of VEGF-A as a migration-activating mediator in miR-200b/200a/429 cluster- or miR-141/200c cluster-transduced MDA-MB-231 cells, a trans-well migration assay was assessed in MCF-7 and MDA-MB-231 cells treated with exogenous VEGF-A protein. The representative crystal violet staining of exogenous VEGF-A (10 ng/ml)-treated MCF-7 and MDA-MB-231 cells that had migrated through the trans-well membranes after 48 h is shown in Fig. 4a. A quantitative analysis of a crystal violet assay showed that exogenous VEGF-A significantly increased MDA-MB-231 cell migration up to 1.50 ± 0.16-fold but had no effect on the migratory ability of MCF-7 cells compared with that of untreated cells (Fig. 4b, untreated vs. VEGF-A, *p* = 0.003). In addition, we investigated whether VEGF-A played a role as a migration-activating mediator in HCC-38 cells. As expected, VEGF-A treatment significantly increased HCC-38 cell migration up to 1.56 ± 0.26-fold (Additional file 7: Figure S7B, untreated vs. VEGF-A, *p* < 0.001).

To verify the exogenous VEGF-A-mediated intracellular signaling pathways responsible for the increased migratory ability of MDA-MB-231 cells, the levels of FAK, AKT, and ERK phosphorylation were evaluated. After cell starvation for 4 h, treatment with exogenous VEGF-A for 15 min, 30 min, 60 min, and 48 h induced the periodic fluctuation of the phosphorylation levels of FAK and AKT and generally increased phosphorylated FAK and AKT, but did not affect the phosphorylation level of ERK in MDA-MB-231 cells (Fig. 4c, d). These data indicated that the activation of the FAK and PI3K/AKT signaling pathways mediated by the exogenous VEGF-A increased the migratory ability of MDA-MB-231 cells. Similar results that VEGF-A stimulated FAK, AKT, and ERK were also observed in HCC-38 cells (Additional file 7: Figure S7C, D).

## Discussion

The role of miR-200 family in regulating the migration and invasion of different cancer cell types is controversial [10, 11]. Moreover, only a small number of miR-200 target genes that regulate cell migration and cancer metastasis has been identified [4, 6], and the mechanisms underlying the functions of miR-200b/200a/429 cluster and miR-141/200c cluster in TNBC cells are not fully understood. It is well-known that the miR-200 family expression levels are significantly lower in highly migratory TNBC cells and metastatic TNBC tumors than other types of breast cancer cells and tumors. In addition, Vrba L et al. reported the repression of miR-200 and miR-141 expression due to aberrant epigenetic gene silencing in aggressive cancer cells, including MDA-MB-231 cells, indicating that the downregulation of miR-200 may contribute to an aggressive TNBC phenotype [17]. ZEB1 and SIP1 have been found to repress primary transcript and mature miR-200 expression in mesenchymal types of breast cancer cells, suggesting a downregulation of miR-200 in TNBC cells through a potential double-negative feedback loop between ZEB1/SIP1 and the miR-200 family [18]. Manav K et al. reported the highest expression of miR-200s in the highly metastatic 4T1 cells, a mouse TNBC cell line, which was consistent with acquisition of epithelial traits in 4T1 cells compared with the weakly metastatic 4TO7 cells. In addition, 4T1 tumors exhibit spontaneous metastasis and colonization of distant organs, which is enhanced by miR-200 overexpression in experimental animal models, furthermore, higher expression of miR-200 levels were found in lung-pleural metastasis samples relative to primary tumor samples in breast cancer patients. These data support the potential role of miR-200s in migration, invasion, metastatic colonization, and metastatic dissemination [6]. Recently, Avery-Kiejda KA et al. found that the miR-200 cluster is upregulated in invasive ductal carcinomas with both lymph node-positive and lymph node-negative TNBC compared with matched normal adjacent tissues [19]. Their reports of in vivo experimental and clinical evidence may indicate that tumor cell populations with increased aggressiveness may have higher miR-200 cluster levels than their less aggressive counterparts within the same TNBC and in normal tissues, the miR-200 cluster, though being generally reduced in TNBC compared with other subtypes, is upregulated in TNBC cells that may support metastatic dissemination. We showed here that the overexpression of miR-200b/200a/429 cluster or miR-141/200c cluster strongly promoted the migration and invasion of MDA-MB-231, HCC-38, and Hs578T cells, typical claudin-low and mesenchymal subtypes of TNBC cell lines [20], compared with those of an ER-positive breast cancer cell line, MCF-7 cells. The migratory and invasive ability of MDA-MB-231 cells was substantially more enhanced in those that overexpressed the miR-141/200c cluster than in those with the miR-200b/200a/429 cluster. These data suggest that the different roles of the miR-200 family members, such as miR-200a, miR-200b, miR-200c, miR-141, and miR-429, on the migration and invasion of different subtypes of human breast cancer cell lines classified by molecular characterization should be further investigated [21].

Dysregulation of the PI3K/AKT signaling pathway has been implicated in mammary carcinogenesis and was suggested to be the mechanism underlying the survival of invasive breast cancer cells [22]. Furthermore, an activated AKT signaling pathway, a common dysregulation observed in breast cancers, has been shown to promote cancer cell growth, survival, and metastasis [23]. FAK is a cytoplasmic tyrosine kinase that plays crucial roles in integrin-mediated signal transduction, and FAK localizes to the sites where transmembrane integrin receptors are clustered to mediate various intracellular signal-transduction pathways [5, 24]. Many recent studies have reported that an increased level of FAK expression highly correlates with the invasiveness and metastasis of human tumors [25–27]. We found that the phosphorylation levels of AKT and FAK in MDA-MB-231 cells were elevated by stable overexpression of the miR-200b/200a/429 cluster and the miR-141/200c cluster. Additionally, increased integrin-αV and phosphorylated FAK co-localized in the transmembrane of miR-200b/200a/429 cluster- and miR-141/200c cluster-transduced MDA-MB-231 cells. These data suggest that the stable overexpression of the 200b/200a/429 cluster and the miR-141/200c cluster in MDA-MB-231 cells may affect the secretion of cytokines or growth factors to activate FAK or the PI3K/AKT signaling pathway. The regulation between the miR-200 family and FAK is not fully understood. A model indicating that a stiffer matrix of breast cancers will activate FAK, which inhibits the miR-200 family and allow for a mesenchymal phenotype has been proposed [28]. This model is not consistent with our observation that the overexpression of the miR-141/200c cluster or the miR-200b/200a/429 cluster increased clustering and expression of integrins and activated FAK and AKT, which regulate cell migration. Our finding proposes a positive cross-talk between FAK and overexpressed miR-200 in TNBC cells.

Many studies have been aimed at understanding the role of cytokines and growth factors in breast cancer progression. Some cytokines and growth factors (IL-1, IL-6, IL-11, TGF-β, and VEGF) stimulate the proliferation and invasion of breast cancer cells, whereas others (IL-12, IL-18, and IFN) suppress breast cancer progression [29]. A recent study reported that miR-200 inhibits angiogenesis by targeting IL-8 and CXCL1 secreted by tumor endothelial and cancer cells [30]. Our study demonstrated that the overexpression of the miR-200b/200a/429 cluster or the miR-141/200c cluster in MDA-MB-231 cells led to a decrease in the secretion of IL-2, IL-4, IL-5, IL-10, IL-13, GM-CSF, INF-γ and TNF-α but a significant increase in the secretion of VEGF-A. VEGF has been reported to activate the PI3K/AKT/forkhead signaling pathway to promote angiogenesis in human endothelial cells [31]. In addition, VEGF-A, the most potent angiogenic factor in tumor angiogenesis, induces oligodendrocyte precursor cell migration through a ROS- and FAK-dependent mechanism [32]. VEGF-A is known to be a direct target of miR-200b [33], but in our results, a significant decrease in VEGF-A levels assessed by western blotting was not observed in miR-200b/200a/429- and miR-141/200c-transduced MDA-MB-231 cells or in miR-141/200c-transduced HCC-38 cells relative to control. Moreover, VEGF-A secretions in MDA-MB-231, HCC-38, and Hs578T cells were increased by the overexpression of the miR-141/200c cluster. The comprehensive interactions between miRNAs and transcription factors (TFs) are expected to comprise “wired” genetic networks to regulate the expression of target genes [34]. In examples of an incoherent feed-forward loop, the direct regulatory effect of TFs on the target gene (VEGF-A) is opposed to the indirect regulatory effect through miR-200 regulation. Taking the comprehensive interactions between miRNAs and protein-coding genes, we propose that miR-200 overexpression in TNBC cells can affect TFs (HIF-1, CREB) or signals (PI3K/AKT) to regulate VEGF-A secretion through an incoherent feed-forward loop. An autocrine loop for VEGF-A to induce breast cancer cell migration/invasion has been well documented [35, 36]. From these reports, we assume that the VEGF-A secreted by miR-200 overexpression interacts with its receptors, such as neuropilin-1 (NP-1) and VEGFR, and stimulates the PI3K/AKT signaling pathway, thus promoting TNBC cell migration and invasion. In this study, we demonstrated that treatment with VEGF-A led to an increase in migratory ability and activated FAK and the PI3K/AKT signaling pathway in MDA-MB-231 cells and HCC-38 cells. Our results strongly support that VEGF-A-mediated FAK or PI3K/AKT signaling pathway modulates cancer cell migration and invasion. By down-regulating miR-200b expression through the PI3K/AKT signaling pathway, the chemokine CCL5 (formerly RANTES) promotes VEGF-dependent angiogenesis in human chondrosarcomas [15]. In addition, synthetic miR-200c downregulates VEGF-A by the direct targeting of the 3’UTR of VEGF-A mRNA in a lung cancer cell line [16]. Contrary to above studies, in this study, the stable overexpression of the miR-200b/200a/429 cluster and the miR-141/200c cluster in MDA-MB-231 cells resulted in increased VEGF-A secretion and induced AKT and FAK phosphorylation. Blocking the PI3K/AKT or FAK signaling pathways using chemical inhibitors inhibited the enhanced migration and invasion in MDA-MB-231 cells overexpressing the miR-200b/200a/429 cluster or the miR-141/200c cluster. Inhibiting the VEGF-A-mediated pathway using anti-VEGF-A-neutralizing antibodies suppressed the elevated AKT and FAK phosphorylation in MDA-MB-231 cells overexpressing miR-141/200c cluster, reversing the enhanced migratory and invasive abilities. These results suggest that the activation of FAK and the PI3K/AKT signaling pathway are directly mediated by elevated VEGF-A secretion, which is involved in the increased migratory and invasive abilities of miR-141/200c cluster-transduced MDA-MB-231 cells. On the other hand, FAK- and PI3K/AKT-independent signaling pathways activated by VEGF-A may lead to an enhanced migratory ability in miR-200b/200a/429 cluster-transduced MDA-MB-231 cells. The present study implies that aberrant expression of miR-200b/200a/429 cluster or the miR-141/200c cluster may play a pro-metastasis role leading to the promotion of migration and invasion of MDA-MB-231 cells.

## Conclusions

The stable overexpression of the miR-141/200c cluster promoted the migratory and invasive ability of TNBC cells through the strong activation of FAK and the PI3K/AKT signaling pathway by increasing VEGF-A secretion compared with the miR-200b/200a/429 cluster.

## Abbreviations

AKT, protein kinase B; FAK, focal adhesion kinase; miR-200, microRNA-200; PI3K, phosphatidylinositol-4,5-bisphosphate 3-kinase; TNBC, triple-negative breast cancer; VEGF, vascular endothelial growth factor

## Additional files

Additional file 1: Figure S1. Comparison of gene expression and migration in miR-200b/200a/429 or miR-141/200c-transduced MCF-7 and MDA-MB-231 cells. **(A)** Fluorescence images of green fluorescent protein in MCF-7 and MDA-MB-231 cells that were transduced using lentivirus encoding both GFP and miR-200 family members. Strong GFP expression was detected in the miR-200 family-transduced cells. Scale bar, 50 μm **(B)** RT-PCR analysis of genes related to epithelial-mesenchymal transition (E-cadherin, fibronectin, vimentin, ZEB1, ZEB2, and snail). E-cadherin expression was induced in the miR-200 family-transduced MDA-MB-231 cells. While snail expression was increased, ZEB1 expression was lower in the miR-200 family-transduced MDA-MB-231 cells than in the non-transduced control cells. **(C)** Images of the crystal violet-stained cells that migrated horizontally in the trans-well migration assay. The stable transduction of miR-200 family slightly suppressed the migratory ability of MCF-7 cells but significantly enhanced the migratory ability of MDA-MB-231 cells. **(D)** Images of the laterally migrated cells as determined using a wound-healing assay. Enhanced lateral migration ability was observed in only the miR-200 family-transduced MDA-MB-231 cells. **(E)** Quantitative analysis of the migratory ability of MCF-7 and MDA-MB-231 cells assessed using trans-well migration assay. The stable transduction of miR-200ab and miR-200c significantly increased the migratory ability of MDA-MB-231 cells but did not influence on the migratory ability of MCF-7 cells. (F) Quantitative analysis of the migratory ability of MCF-7 and MDA-MB-231 cells assessed using wound-healing assays. The stable transduction of miR-200ab led to a significant increase in lateral migration of MDA-MB-231 cells. All experiments were performed at least in triplicate, and the values are the mean values ± standard deviation. **p* < 0.05. (JPG 292 kb) Additional file 2: Figure S2. Flow cytometric analysis of GFP in miR-200b/200a/429 or miR-141/200c-transduced MCF-7 and MDA-MB-231 cells. Flow cytometry analysis of the percentage of GFP-positive cells among the miR-200ab- and miR-200c-transduced MCF-7 and MDA-MB-231 cells. GFP-positive cells among the miR-200 family-transduced MCF-7 and MDA-MB-231 cells were found to be greater than 90 %. (JPG 118 kb) Additional file 3: Figure S3. Migration in miR-141/200c-transduced MDA-MB-231 cells. Quantitative analysis of the migratory ability in of MDA-MB-231 and miR-141/200c-transduced MDA-MB-231 cells was performed in trans-well migration assay with 10 % FBS in the lower chamber. The migratory ability of the miR-200c cells (~1.5-fold) was significantly increased compared with those of the control cells. All experiments were performed at least in triplicate, and the values are the mean values ± standard deviation. ***p* < 0.001. (JPG 14 kb) Additional file 4: Figure S4. Migration in miR-141/200c-transduced HCC-38 and Hs578T cells treated with an anti-VEGF-A-neutralizing antibody. (**A, D**) Migration in miR-141/200c-transduced HCC-38 and Hs578T cells. Images of the crystal violet-stained cells that migrated horizontally in the trans-well migration assay (upper). The absorbance values of extracted crystal violet in migrated cells (lower). The migratory abilities of the miR-200c cells (~1.6-fold and ~1.7-fold, HCC-38 and Hs578T, respectively) were significantly increased compared with those of the control cells. **(B, E)** Measurement of the secreted levels of cytokines and growth factors (IL-2, IL-4, IL-5, IL-10, IL-12, IL-13, GM-CSF, IFN-γ, TNF-α, and VEGF-A). Transduction of miR-141/200c into HCC-38 and Hs578T cells promoted significantly higher VEGF-A secretion than that of control cells. **(C, F)** Trans-well migration of anti-VEGF-A-neutralizing antibody-treated cells. The enhanced migration of the miR-141/200c-transduced HCC-38 cells were significantly suppressed by treatment with anti-VEGF-A-neutralizing antibodies, but miR-141/200c-transduced Hs578T cells still showed increased migratory ability compared with control cells. **p* < 0.05, ***p* < 0.001. (JPG 188 kb) Additional file 5: Figure S5. microRNA expression levels of miR-200 cluster transduced MCF-7, MDA-MB-231, HCC-38, and Hs578T cells. Quantitative real-time RT-PCR of microRNAs (miR-200a, miR-200b, and miR-200c). **(A, B, C)** microRNAs in MCF-7 cells. The miR-200ab cells transduced with the miR-200b/200a/429 cluster exhibited high levels of expression of miR-200a (~60-fold) and miR-200b (~40-fold) relative to those of the control cells. The miR-200c cells transduced with the miR-200c/141 cluster exhibited remarkably high levels of expression of miR-200c (~266-fold) relative to those of the control cells. **(D, E, F)** microRNAs in MDA-MB-231. The miR-200ab cells transduced with the miR-200b/200a/429 cluster exhibited high expression levels of miR-200a (~6-fold) and miR-200b (~40-fold) relative to those of the control cells. The miR-200c cells transduced with the miR-200c/141 cluster exhibited remarkably high expression levels of miR-200c (~93-fold) relative to those of the control cells. **(G)** microRNAs in HCC-38. The miR-200c cells transduced with the miR-200c/141 cluster exhibited remarkably high expression levels of miR-200c (~252-fold) relative to those of the control cells. **(H)** microRNAs in Hs578T. The miR-200c cells transduced with the miR-200c/141 cluster exhibited remarkably high expression levels of miR-200c (~205-fold) relative to those of the control cells. **p* < 0.05, ***p* < 0.001. (JPG 50 kb) Additional file 6: Figure S6. VEGF-A expression and signaling pathways associated with enhanced migration in miR-141/200c-transduced HCC-38 cells. **(A)** Representative image of western blotting analysis of VEGF-A levels. β − actin was used as an internal reference. **(B)** Densitometric quantification of VEGF-A in the miR-141/200c-transduced cells relative to the control cells. **(C)** Western blotting analysis of the levels of phosphorylated FAK, AKT, ERK, and integrin-αV expression. The levels of FAK and AKT phosphorylation were significantly higher in the miR-141/200c-transduced cells than in the control cells, but the level of ERK phosphorylation was similar between the miR-141/200c-transduced cells and control cells. The miR-141/200c-transduction also increased the level of integrin-αV expression. β − actin was used as an internal reference. **(D)** Densitometric quantification of FAK, AKT, and ERK phosphorylation in the miR-141/200c-transduced cells relative to the control cells. **(E)** Immunofluorescence analysis of phosphorylated FAK and integrin-αV. Phosphorylated FAK and integrin-αV were overexpressed and highly localized at membrane surfaces in the miR-141/200c-transduced cells. **p* < 0.05. Scale bar, 10 μm. (JPG 102 kb) Additional file 7: Figure S7. Migration and signaling pathways in VEGF-A-stimulated HCC-38 cells. **(A)** Migration in VEGF-A-stimulated HCC-38 cells. Images of the crystal violet-stained cells that migrated horizontally in the trans-well migration assay. **(B)** The absorbance values of extracted crystal violet in migrated cells (lower). Migration of the VEGF-A-treated HCC-38 cells (~1.6-fold) was enhanced compared with that of the untreated control cells. **(C)** Representative image of western blotting of phosphorylated AKT, FAK and ERK and total AKT, FAK and ERK in HCC-38 cells treated with VEGF-A. **(D)** Densitometric quantification of VEGF-A in the miR-141/200c-transduced cells relative to the control cells. β − actin was used as an internal reference. The levels of FAK, AKT, and ERK phosphorylation were increased in the VEGF-A-treated HCC-38 cells. **p* < 0.05, ***p* < 0.001. (JPG 108 kb)
