# Supplementary figures and images for: Overexpression of the miR-141/200c cluster promotes the migratory and invasive ability of triple-negative breast cancer cells through the activation of the FAK and PI3K/AKT signaling pathways by secreting VEGF-A
Source: BMC Cancer. 2016 Aug 2;16:570. doi: 10.1186/s12885-016-2620-7 (PMC4969651; doi:10.1186/s12885-016-2620-7)

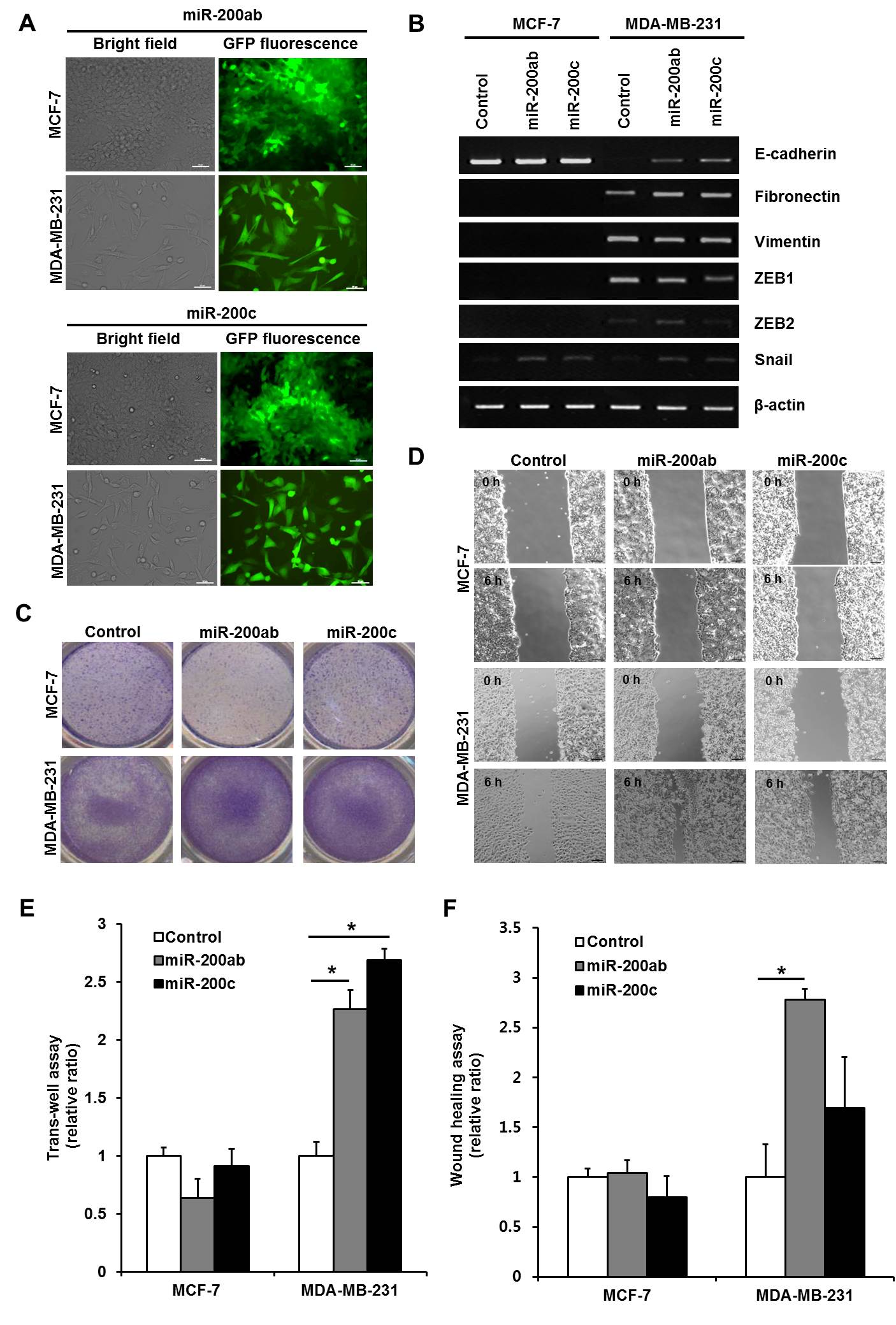

Supplement: Additional file 1: Figure S1. — Comparison of gene expression and migration in miR-200b/200a/429 or miR-141/200c-transduced MCF-7 and MDA-MB-231 cells. (A) Fluorescence images of green fluorescent protein in MCF-7 and MDA-MB-231 cells that were transduced using lentivirus encoding both GFP and miR-200 family members. Strong GFP expression was detected in the miR-200 family-transduced cells. Scale bar, 50 μm (B) RT-PCR analysis of genes related to epithelial-mesenchymal transition (E-cadherin, fibronectin, vimentin, ZEB1, ZEB2, and snail). E-cadherin expression was induced in the miR-200 family-transduced MDA-MB-231 cells. While snail expression was increased, ZEB1 expression was lower in the miR-200 family-transduced MDA-MB-231 cells than in the non-transduced control cells. (C) Images of the crystal violet-stained cells that migrated horizontally in the trans-well migration assay. The stable transduction of miR-200 family slightly suppressed the migratory ability of MCF-7 cells but significantly enhanced the migratory ability of MDA-MB-231 cells. (D) Images of the laterally migrated cells as determined using a wound-healing assay. Enhanced lateral migration ability was observed in only the miR-200 family-transduced MDA-MB-231 cells. (E) Quantitative analysis of the migratory ability of MCF-7 and MDA-MB-231 cells assessed using trans-well migration assay. The stable transduction of miR-200ab and miR-200c significantly increased the migratory ability of MDA-MB-231 cells but did not influence on the migratory ability of MCF-7 cells. (F) Quantitative analysis of the migratory ability of MCF-7 and MDA-MB-231 cells assessed using wound-healing assays. The stable transduction of miR-200ab led to a significant increase in lateral migration of MDA-MB-231 cells. All experiments were performed at least in triplicate, and the values are the mean values ± standard deviation. *p < 0.05. (JPG 292 kb) [file 12885_2016_2620_MOESM1_ESM.jpg]

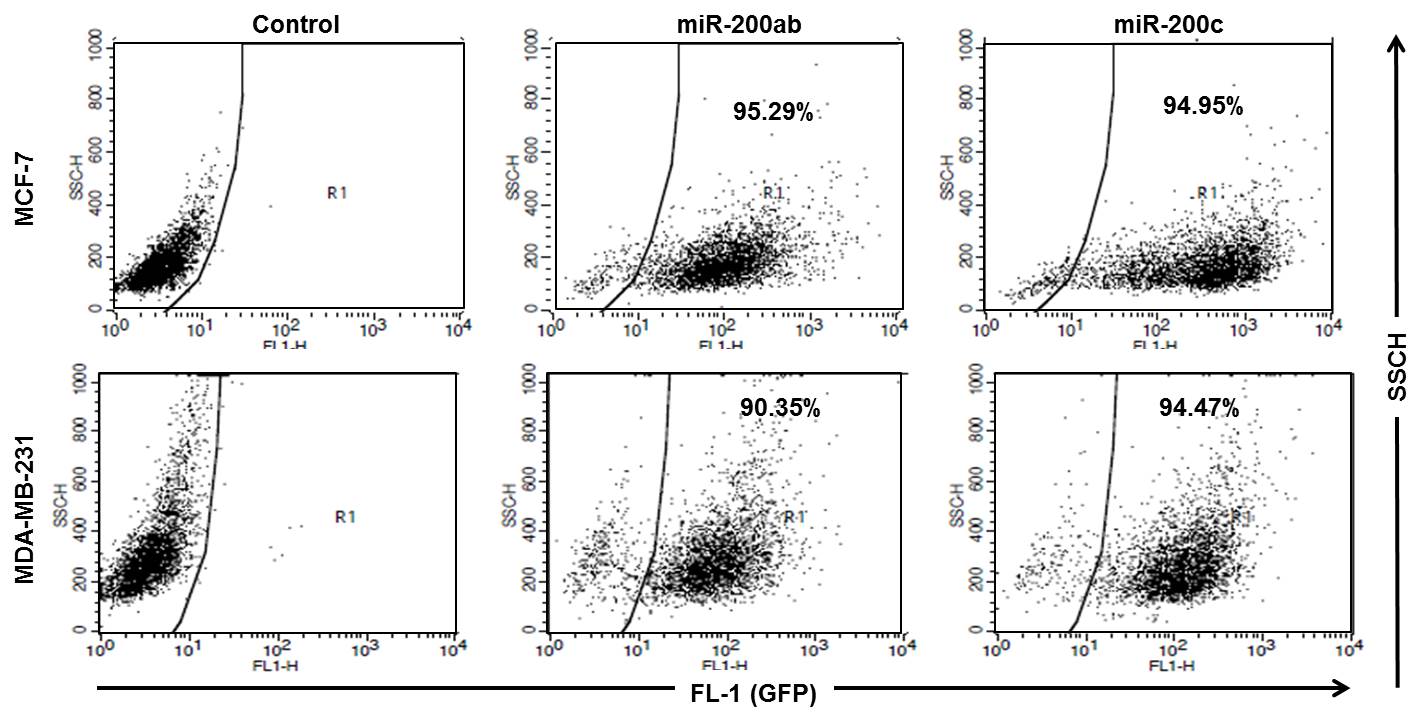

Supplement: Additional file 2: Figure S2. — Flow cytometric analysis of GFP in miR-200b/200a/429 or miR-141/200c-transduced MCF-7 and MDA-MB-231 cells. Flow cytometry analysis of the percentage of GFP-positive cells among the miR-200ab- and miR-200c-transduced MCF-7 and MDA-MB-231 cells. GFP-positive cells among the miR-200 family-transduced MCF-7 and MDA-MB-231 cells were found to be greater than 90 %. (JPG 118 kb) [file 12885_2016_2620_MOESM2_ESM.jpg]

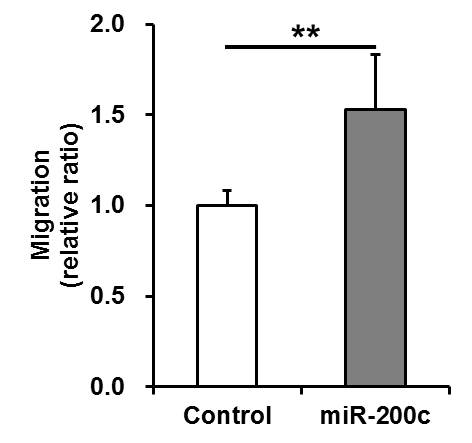

Supplement: Additional file 3: Figure S3. — Migration in miR-141/200c-transduced MDA-MB-231 cells. Quantitative analysis of the migratory ability in of MDA-MB-231 and miR-141/200c-transduced MDA-MB-231 cells was performed in trans-well migration assay with 10 % FBS in the lower chamber. The migratory ability of the miR-200c cells (~1.5-fold) was significantly increased compared with those of the control cells. All experiments were performed at least in triplicate, and the values are the mean values ± standard deviation. **p < 0.001. (JPG 14 kb) [file 12885_2016_2620_MOESM3_ESM.jpg]

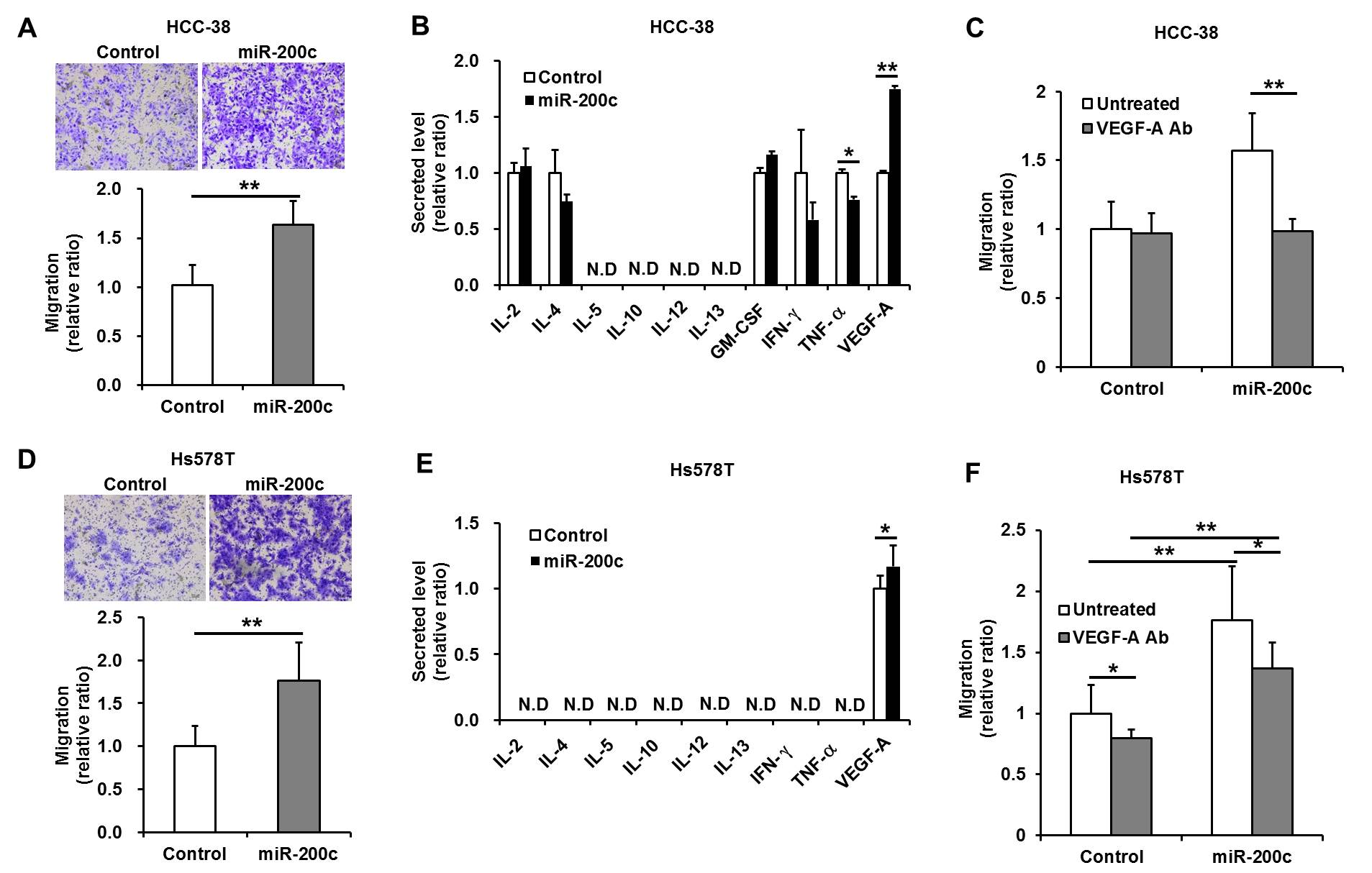

Supplement: Additional file 4: Figure S4. — Migration in miR-141/200c-transduced HCC-38 and Hs578T cells treated with an anti-VEGF-A-neutralizing antibody. (A, D) Migration in miR-141/200c-transduced HCC-38 and Hs578T cells. Images of the crystal violet-stained cells that migrated horizontally in the trans-well migration assay (upper). The absorbance values of extracted crystal violet in migrated cells (lower). The migratory abilities of the miR-200c cells (~1.6-fold and ~1.7-fold, HCC-38 and Hs578T, respectively) were significantly increased compared with those of the control cells. (B, E) Measurement of the secreted levels of cytokines and growth factors (IL-2, IL-4, IL-5, IL-10, IL-12, IL-13, GM-CSF, IFN-γ, TNF-α, and VEGF-A). Transduction of miR-141/200c into HCC-38 and Hs578T cells promoted significantly higher VEGF-A secretion than that of control cells. (C, F) Trans-well migration of anti-VEGF-A-neutralizing antibody-treated cells. The enhanced migration of the miR-141/200c-transduced HCC-38 cells were significantly suppressed by treatment with anti-VEGF-A-neutralizing antibodies, but miR-141/200c-transduced Hs578T cells still showed increased migratory ability compared with control cells. *p < 0.05, **p < 0.001. (JPG 188 kb) [file 12885_2016_2620_MOESM4_ESM.jpg]

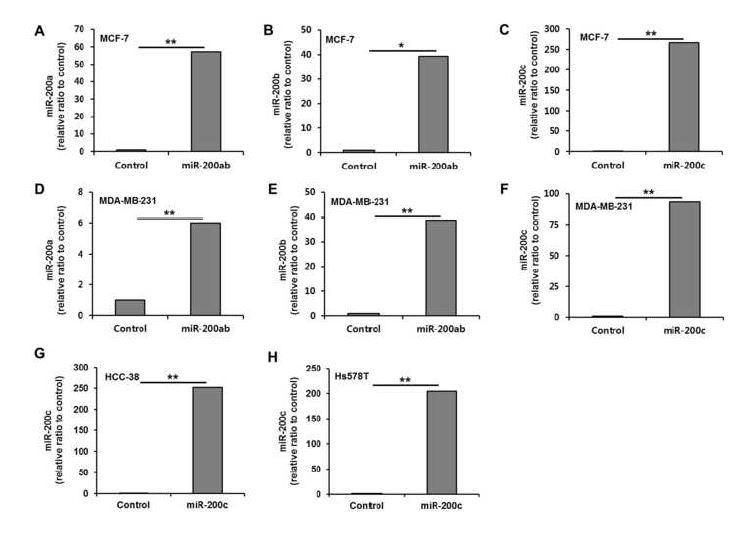

Supplement: Additional file 5: Figure S5. — microRNA expression levels of miR-200 cluster transduced MCF-7, MDA-MB-231, HCC-38, and Hs578T cells. Quantitative real-time RT-PCR of microRNAs (miR-200a, miR-200b, and miR-200c). (A, B, C) microRNAs in MCF-7 cells. The miR-200ab cells transduced with the miR-200b/200a/429 cluster exhibited high levels of expression of miR-200a (~60-fold) and miR-200b (~40-fold) relative to those of the control cells. The miR-200c cells transduced with the miR-200c/141 cluster exhibited remarkably high levels of expression of miR-200c (~266-fold) relative to those of the control cells. (D, E, F) microRNAs in MDA-MB-231. The miR-200ab cells transduced with the miR-200b/200a/429 cluster exhibited high expression levels of miR-200a (~6-fold) and miR-200b (~40-fold) relative to those of the control cells. The miR-200c cells transduced with the miR-200c/141 cluster exhibited remarkably high expression levels of miR-200c (~93-fold) relative to those of the control cells. (G) microRNAs in HCC-38. The miR-200c cells transduced with the miR-200c/141 cluster exhibited remarkably high expression levels of miR-200c (~252-fold) relative to those of the control cells. (H) microRNAs in Hs578T. The miR-200c cells transduced with the miR-200c/141 cluster exhibited remarkably high expression levels of miR-200c (~205-fold) relative to those of the control cells. *p < 0.05, **p < 0.001. (JPG 50 kb) [file 12885_2016_2620_MOESM5_ESM.jpg]

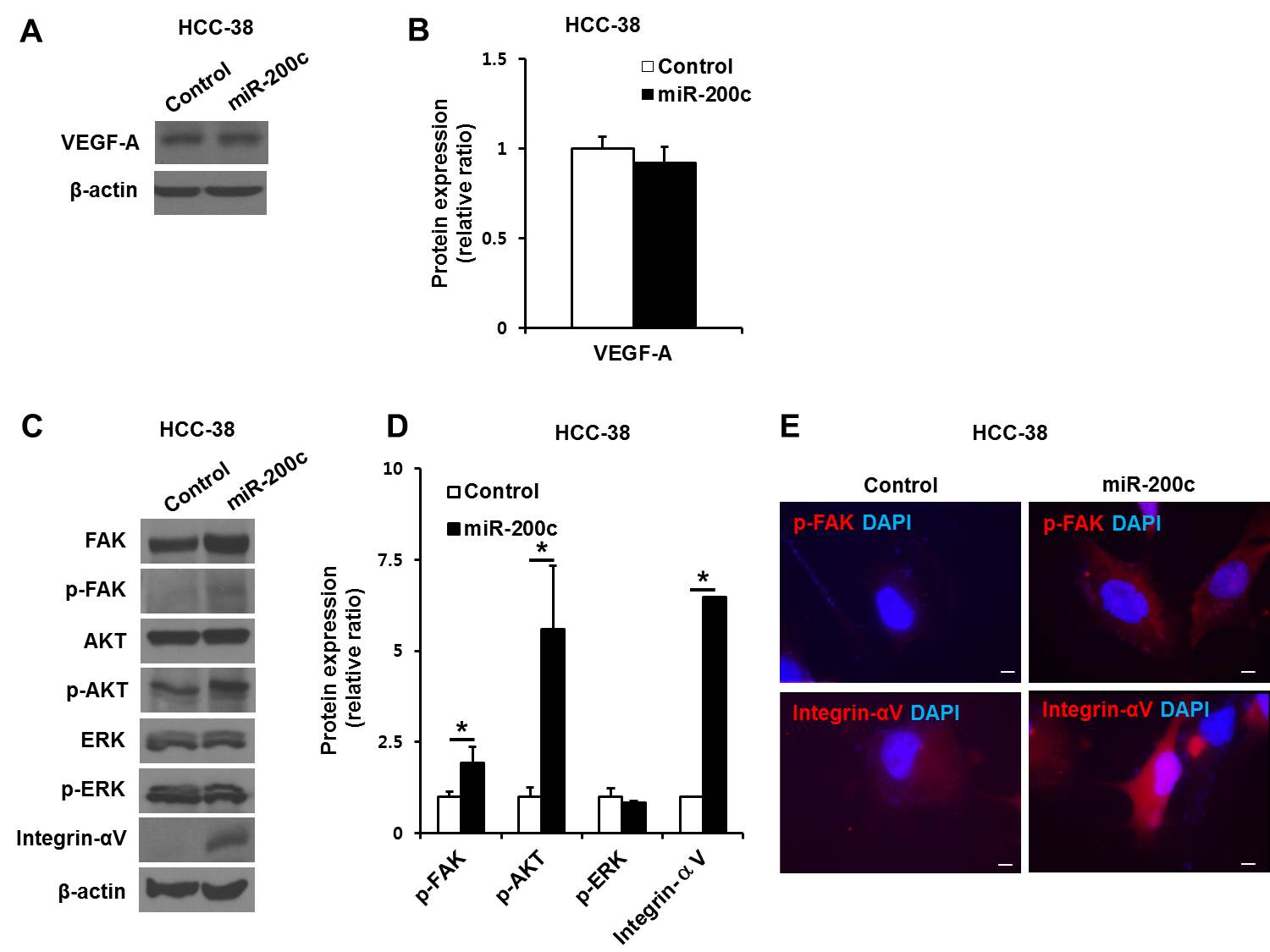

Supplement: Additional file 6: Figure S6. — VEGF-A expression and signaling pathways associated with enhanced migration in miR-141/200c-transduced HCC-38 cells. (A) Representative image of western blotting analysis of VEGF-A levels. β − actin was used as an internal reference. (B) Densitometric quantification of VEGF-A in the miR-141/200c-transduced cells relative to the control cells. (C) Western blotting analysis of the levels of phosphorylated FAK, AKT, ERK, and integrin-αV expression. The levels of FAK and AKT phosphorylation were significantly higher in the miR-141/200c-transduced cells than in the control cells, but the level of ERK phosphorylation was similar between the miR-141/200c-transduced cells and control cells. The miR-141/200c-transduction also increased the level of integrin-αV expression. β − actin was used as an internal reference. (D) Densitometric quantification of FAK, AKT, and ERK phosphorylation in the miR-141/200c-transduced cells relative to the control cells. (E) Immunofluorescence analysis of phosphorylated FAK and integrin-αV. Phosphorylated FAK and integrin-αV were overexpressed and highly localized at membrane surfaces in the miR-141/200c-transduced cells. *p < 0.05. Scale bar, 10 μm. (JPG 102 kb) [file 12885_2016_2620_MOESM6_ESM.jpg]

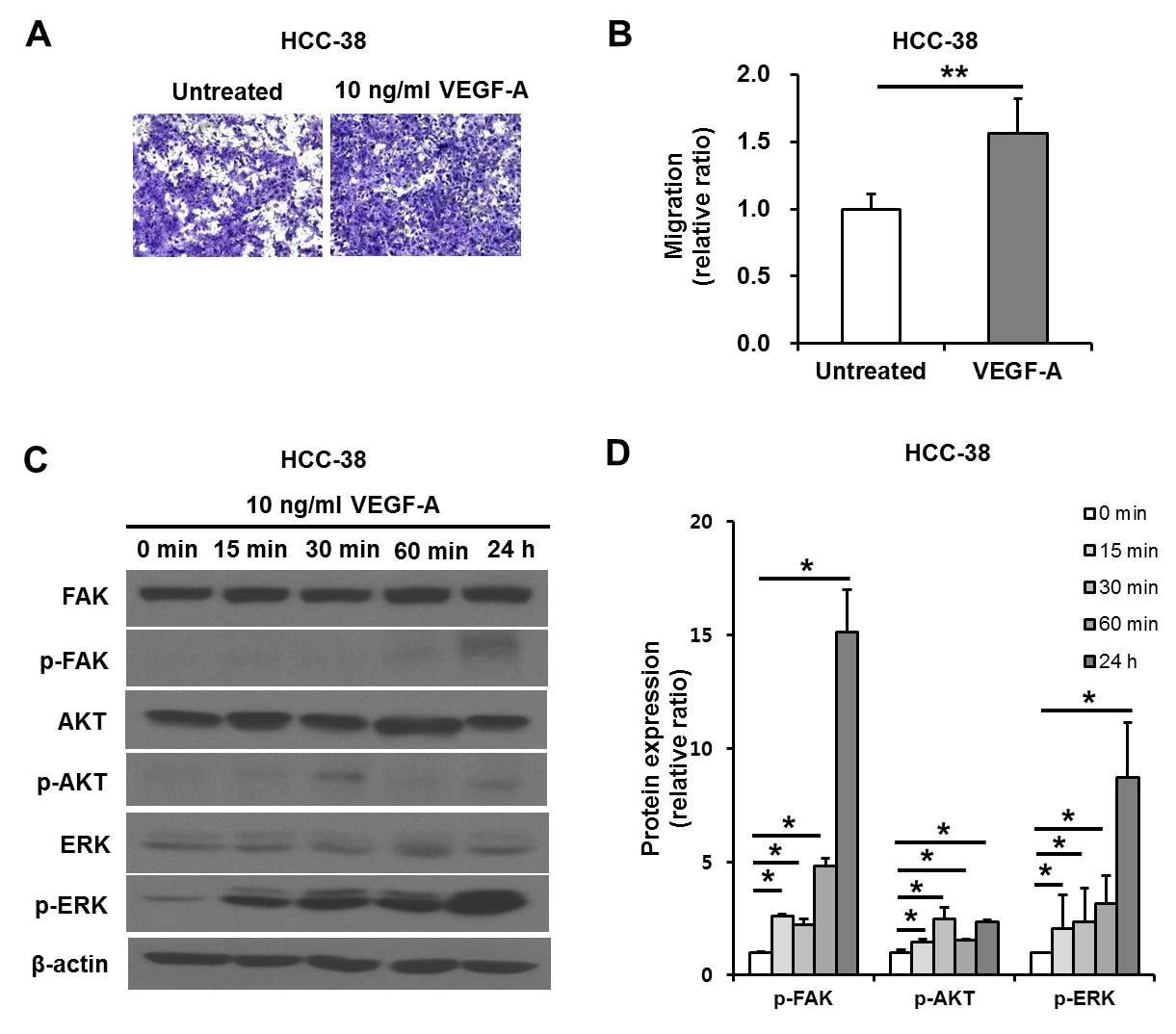

Supplement: Additional file 7: Figure S7. — Migration and signaling pathways in VEGF-A-stimulated HCC-38 cells. (A) Migration in VEGF-A-stimulated HCC-38 cells. Images of the crystal violet-stained cells that migrated horizontally in the trans-well migration assay. (B) The absorbance values of extracted crystal violet in migrated cells (lower). Migration of the VEGF-A-treated HCC-38 cells (~1.6-fold) was enhanced compared with that of the untreated control cells. (C) Representative image of western blotting of phosphorylated AKT, FAK and ERK and total AKT, FAK and ERK in HCC-38 cells treated with VEGF-A. (D) Densitometric quantification of VEGF-A in the miR-141/200c-transduced cells relative to the control cells. β − actin was used as an internal reference. The levels of FAK, AKT, and ERK phosphorylation were increased in the VEGF-A-treated HCC-38 cells. *p < 0.05, **p < 0.001. (JPG 108 kb) [file 12885_2016_2620_MOESM7_ESM.jpg]
